# An Injectable and Photocrosslinkable Hydrogel Based on Recombinant Human Collagen Type XVII Promotes Osteochondral Regeneration by Activating the TGF β/SMAD Signaling Pathway

**DOI:** 10.34133/bmr.0412

**Published:** 2026-09-14

**Authors:** Guanhao Yang, Wei Li, YanEn Wang, Xin Zhang, Haihang Li, Tinglin Zhang, Denghui Liu, Anzhao Wang, Jie Gao, Zhongtang Liu, Yu Sun, Xuan Huang

**Affiliations:** ^1^ Department of Orthopedics, the First Affiliated Hospital of Naval Medical University, Shanghai 200433, China.; ^2^ Suzhou Institute of Biomedical Engineering and Technology, Chinese Academy of Sciences, Suzhou 215163, Jiangsu, China.; ^3^ School of Biomedical Engineering (Suzhou), Division of Life Sciences and Medicine, University of Science and Technology of China, Hefei 230026, Anhui, China.; ^4^ Department of Burn Surgery, the First Affiliated Hospital of Naval Medical University, Shanghai 200433, China.; ^5^ Department of Stomatology, the First Affiliated Hospital of Naval Medical University, Shanghai 200433, China.; ^6^ Jiangsu Trautec Medical Technology Co., Ltd., Changzhou 213100, Jiangsu, China.; ^7^ Changhai Clinical Research Unit, the First Affiliated Hospital of Naval Medical University, Shanghai 200433, China.; ^8^ Department of Orthopedics, the Guanghua Hospital of Integrated Traditional Chinese and Western Medicine, Shanghai 200052, China.; ^9^ Department of Otolaryngology-Head & Neck Surgery, Shanghai Tenth People’s Hospital, School of Medicine, Tongji University, Shanghai 200072, China.

## Abstract

The management of articular cartilage defects remains a major clinical challenge owing to the tissue’s limited intrinsic regenerative capacity. Conventional collagen-based hydrogels often fail to achieve functional repair due to a mismatch between their biological functions and mechanical properties. Here, for the first time, we apply recombinant human collagen type XVII (rhCOL17) to cartilage repair by engineering an injectable and photocrosslinkable hydrogel based on rhCOL17. A systematic screening identified the 15% methacrylated rhCOL17 (CMA) formulation as possessing optimal mechanical properties and biocompatibility, featuring a suitable 3-dimensional porous structure, moderate swelling, slow degradation, and rapid in situ photocrosslinking capability. In vitro experiments demonstrated that the CMA hydrogel is noncytotoxic and notably promotes the proliferation, migration, and chondrogenic differentiation of bone marrow-derived mesenchymal stem cells (BMSCs) and chondrocytes, as evidenced by the up-regulation of SOX9, COL2, and ACAN. In vivo, using a rat knee joint full-thickness cartilage defect model, the BMSCs-laden 15% CMA composite hydrogel (BMSCs@CMA) achieved superior osteochondral integrated repair, with the regenerated tissue closely resembling native cartilage in macroscopic morphology, subchondral bone mineralization, histological structure, and collagen type II deposition. Mechanistically, transcriptome sequencing, molecular docking, and molecular dynamics simulations collectively demonstrate that rhCOL17 directly binds to transforming growth factor-β1 (TGF-β1) with high affinity and stable interaction, subsequently activating the TGF-β/SMAD signaling pathway to orchestrate BMSCs chondrogenesis. In conclusion, this study establishes the pivotal role of collagen type XVII in cartilage regeneration, presenting a promising, minimally invasive strategy with mechanical adaptability and active inductive functionality for functional cartilage defect repair, and expands the cross-tissue application prospects of rhCOL17.

## Introduction

Articular cartilage defects, which are commonly caused by acute trauma, chronic degenerative joint disease, and age-related degeneration, often lead to persistent joint pain and progressive motor dysfunction and eventually irreversible end-stage osteoarthritis [1]. Owing to the absence of vascular, lymphatic, and nerve innervation, as well as the extremely slow metabolic turnover of the extracellular matrix (ECM), cartilage tissue has a negligible intrinsic self-repair capacity, making functional cartilage restoration a long-standing unmet need in clinical orthopedics and sports medicine [2]. Current mainstream therapies such as microfracture surgery and autologous chondrocyte transplantation can temporarily relieve symptoms, but the regenerated tissue is mostly fibrocartilage with poor mechanical properties and suffers from long-term degeneration and poor tissue integration, failing to achieve functional cartilage repair [3]. Injectable hydrogels have become a research focus in the field of cartilage tissue regeneration because of their advantages of precise filling of irregular defects, minimally invasive delivery, and construction of biomimetic microenvironments [4]. Among them, collagen-based hydrogels are the preferred scaffold materials for cartilage repair owing to their high degree of biomimicry with natural ECM and exhibit notable advantages in terms of biocompatibility, controllable degradability, and nutrient permeability [5].

Collagen, the most abundant structural protein in the human ECM, is widely distributed in connective tissues such as cartilage, bone, and tendons [6]. In articular cartilage, collagen type II (COL2) accounts for 95% of the total collagen content and is a characteristic component of cartilage tissue; its inherent advantages in chondrogenesis have been confirmed [7]. However, the separation and purification of COL2 are complex; its sources are scarce, and it tends to induce fibrocartilage formation, which greatly restricts its clinical application [8]. Collagen Type I (COL1) has a wide range of sources and mature preparation technologies and has become widely used as a collagen material in cartilage engineering [9]. Nevertheless, hydrogels constructed from COL1 often suffer from surface splitting, fibrosis, and poor integration with surrounding cartilage tissue after implantation, affecting the long-term stability of the repaired tissue [10]. Collagen composite biomaterials prepared with inorganic ceramics or conductive polymers have prominent technical flaws. The majority of these scaffolds rely on high-temperature curing or toxic photoinitiators that impair bioactive cargos, and they lack inherent chondrogenic signals to guide stem cell differentiation. Such materials only provide passive mechanical support and cannot achieve targeted cartilage regeneration regulation [11–13]. Beyond scaffold-based strategies, hydrogels loaded with only a single bioactive factor also exhibit limited anti-inflammatory capacity and face translational challenges in rodent pre-clinical studies. In addition, stand-alone magnetoelectric stimulation materials lack chondrocyte-targeted electroregulatory cues and integrated cartilage scaffold designs. These limitations highlight the unaddressed gap for multifunctional composites that combine bioactive factor delivery, inflammation modulation, and wireless bioelectric stimulation [14,15]. It is thus evident that conventional scaffolds are plagued by mismatched biological functions and mechanical properties, and novel collagen-based scaffold materials that combine robust mechanical adaptability, active biological function, and excellent tissue integration capacity for cartilage repair are urgently needed.

In this context, the functional diversity of collagen type XVII (COL17) provides a new research perspective for cartilage tissue regeneration. COL17 was originally discovered in the skin as a core component of hemidesmosomes, maintaining the integrity of the epidermis–dermis junction [16]. It also regulates the quiescence and self-renewal of hair follicle stem cells [17] and participates in the regulation of cell proliferation, migration, and differentiation through its cell adhesion sites and signal transduction domains [18]. Although current research on COL17 is focused mainly on the skin [19], its core functions, such as tissue interface maintenance, stem cell niche regulation, and cell signal transduction, precisely address the pain points of cartilage repair, including poor scaffold–tissue integration, insufficient regulation of seed cell differentiation, and the passive support of traditional scaffolds. Moreover, the cross-tissue adaptability of COL17 makes it a potentially ideal material for cartilage regeneration. However, the content of COL17 in natural tissues is extremely low, and its extraction and purification are difficult [20]. Recombinant human collagen type XVII (rhCOL17), a recombinant product retaining the core active fragments of natural COL17, not only solves the source problem of natural COL17 but also has the advantages of high purity, low immunogenicity, and controllable batch-to-batch consistency, laying a solid material foundation for its application in tissue engineering [21].

Insufficient quantity and low activity of endogenous mesenchymal stem cells in the cartilage defect area are other key factors that restrict self-repair [22]. Bone marrow-derived mesenchymal stem cells (BMSCs) are ideal seed cells for cartilage regeneration because of their easy isolation, expansion, and chondrogenic differentiation potential [23]. However, direct implantation into the defect area is associated with a low cell retention rate, insufficient survival, and uncontrollable differentiation [24]. Injectable hydrogels can deliver BMSCs to the defect area precisely in a minimally invasive manner, provide a 3-dimensional (3D) biomimetic microenvironment for them, and effectively overcome the limitations of simple cell therapy [25]. On this basis, constructing a composite hydrogel containing BMSCs and rhCOL17 by combining the functional properties of rhCOL17 with the carrier advantages of hydrogels is expected to achieve synergistic repair of cartilage defects through “material scaffold-seed cell-biological signal”.

**Fig. 1.**
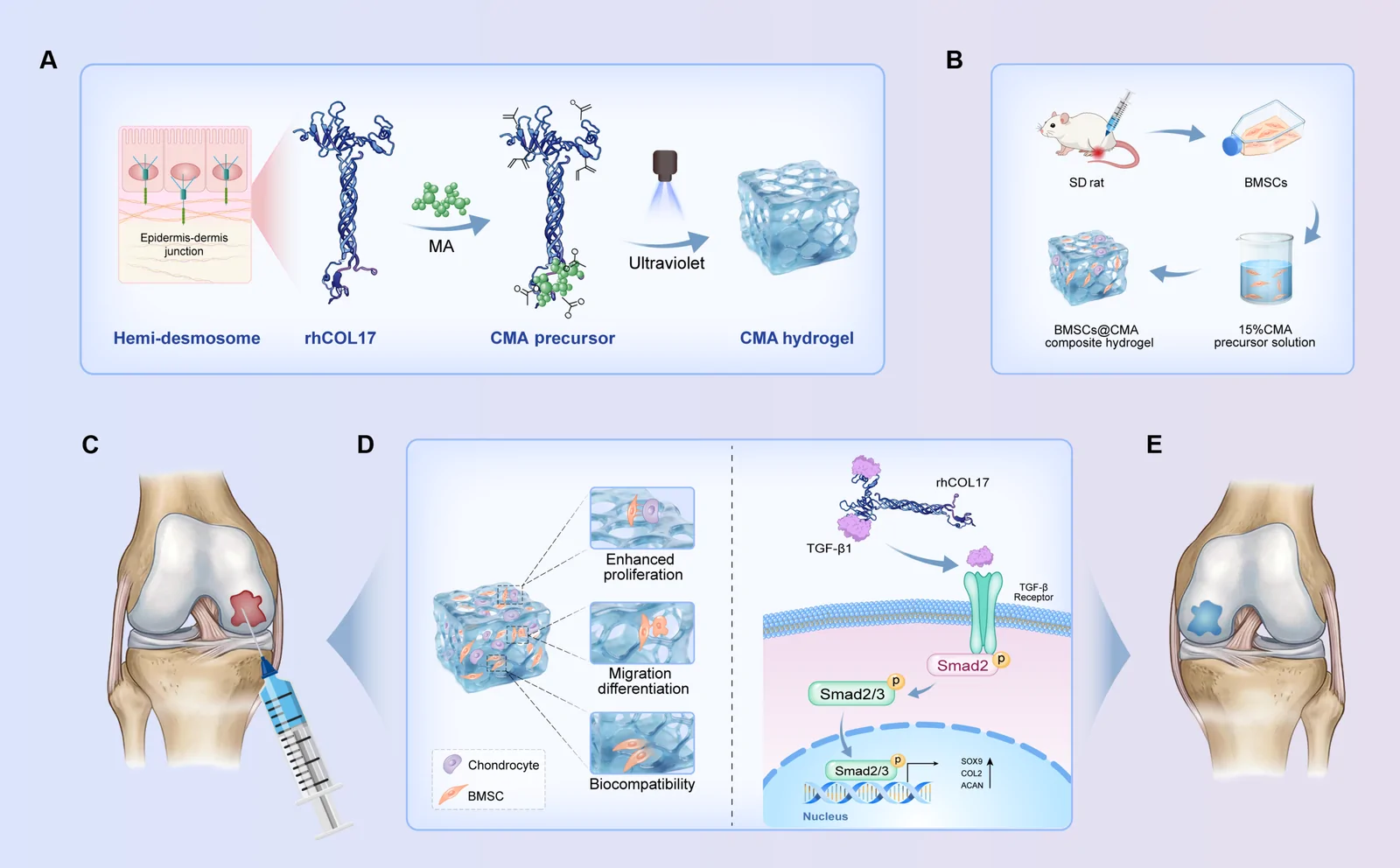
Schematic of the fabrication, in vivo application, and repair mechanism of the injectable and photocrosslinkable hydrogel based on recombinant human collagen type XVII (rhCOL17). (A) Preparation of methacrylated rhCOL17 (CMA) hydrogel via methacrylic anhydride (MA) modification and ultraviolet photocrosslinking using rhCOL17 as the raw material. (B) Loading rat bone marrow-derived mesenchymal stem cells (BMSCs) to construct the BMSCs@CMA composite hydrogel. (C) Minimally invasive injection and implantation into a rat knee joint full-thickness cartilage defect model. (D) The composite hydrogel promotes cell proliferation, migration, and chondrogenic differentiation and regulates cartilage-specific gene expression by activating the TGF-β/SMAD signaling pathway. (E) Functional repair of cartilage defect is ultimately achieved.

To address these challenges, we herein designed and engineered a BMSCs-laden, injectable and photocrosslinkable hydrogel based on rhCOL17. Specifically, we synthesized a methacrylated rhCOL17 (CMA) to construct an injectable hydrogel system. The mechanical properties and biocompatibility of CMA were systematically optimized. We then investigated the therapeutic efficacy of the BMSCs-laden 15% CMA composite hydrogel (BMSCs@CMA) in a rat knee joint full-thickness cartilage defect model and probed the underlying molecular mechanism. This study innovatively introduces rhCOL17 into cartilage repair systems. Via mild cytocompatible photocrosslinking, the fabricated hydrogel is equipped with endogenous chondrogenic cues. The transforming growth factor-β (TGF-β)/SMAD-mediated chondrogenic regulatory mechanism of rhCOL17 is systematically elucidated herein, and a minimally invasive, clinically translatable hydrogel platform integrating bioactive collagen and mesenchymal stem cells is constructed to achieve integrated osteochondral reconstruction. This research addresses the unfulfilled demand for cartilage-targeted recombinant collagen biomaterials and clarifies the core regulatory role of rhCOL17 for the first time, providing novel design principles for the development of collagen-based tissue engineering scaffolds (Fig. 1).

## Materials and Methods

### Cell isolation and culture

All animal experimental procedures were approved by the Ethics Committee of the First Affiliated Hospital of Naval Medical University (Approval No. 2025-050, Shanghai, China). Newborn Sprague–Dawley (SD) rats were anesthetized and sacrificed, and the femurs and tibias were aseptically separated. The bone marrow cavity was flushed with Dulbecco’s Modified Eagle Medium (DMEM; Thermo Fisher Scientific, USA), and the cell suspension was collected. After purification by density gradient centrifugation, the cells were inoculated into DMEM supplemented with 10% fetal bovine serum (Solarbio, China) and cultured at 37 °C with 5% CO_2_.

Cartilage tissue from SD rats was aseptically separated, washed with phosphate-buffered saline (PBS), minced, and digested overnight in DMEM/F12 (Thermo Fisher Scientific, USA) supplemented with 0.2% type II collagenase (Macklin, China) and 1% penicillin–streptomycin (Biosharp, China) at 37 °C. The cell suspension was collected, filtered, and centrifuged, and the precipitated cells were resuspended in DMEM/F12 medium and cultured at 37 °C with 5% CO_2_.

### Preparation and characterization of CMA

#### Preparation of CMA

The rhCOL17 was purchased from Jiangsu Chuangjian Medical Technology Co., Ltd. and its core raw material data are provided in the Supplementary Materials (Table S1). The synthesis of CMA was performed on the basis of an optimized existing method [26]. The specific steps were as follows: 5 g of rhCOL17 sponge was dissolved in 50 ml of PBS, 1 ml of methacrylic anhydride (MA) was slowly added dropwise to the above solution within 10 min, and the pH was adjusted to 8.5 with 1 M sodium hydroxide solution, followed by a constant-temperature reaction at 60 °C for 4 h. Afterwards, the product was put into a dialysis bag with a molecular weight cutoff of 14 kDa for dialysis for 3 d and freeze-dried to obtain the CMA. The prepared CMA sponge was stored in a refrigerator at 4 °C, protected from light and moisture.

#### Characterization of CMA

Firstly, 20 mg of rhCOL17 or CMA was dissolved in 1 ml of D_2_O, and a ^1^H nuclear magnetic resonance (^1^H NMR) spectrometer (Bruker, Germany) was used to detect the changes in the molecular structure of the CMA before and after the grafting reaction. The spectral range of the Fourier transform infrared (FTIR) spectrometer (Shimadzu, Japan) was set to 400 to 4,000 cm^−1^. After background subtraction, the attenuated total reflectance (ATR) correction was applied, and the infrared absorption spectrum of the sample was drawn to detect the changes in the chemical functional groups of the CMA before and after the grafting reaction. The time-of-flight mass spectrometry (TOF-MS) system (AB SCIEX, USA) was used to detect the relative molecular weight of CMA before and after the grafting reaction in positive ion mode with a linear method.

### Microstructure observation

Cylindrical molds were used to prepare hydrogel samples with a diameter of 11.2 mm and a height of 4 mm, which were then freeze-dried. Subsequently, the samples were gold-sputtered, and their cross-sectional morphology was observed via scanning electron microscopy (SEM; ZEISS, Germany) at a voltage of 2 kV. ImageJ software (Wayne Rasband, USA) was utilized to determine pore size and porosity based on the obtained SEM images.

### Rheology and compression tests

Cylindrical molds were used to prepare hydrogel samples with a diameter of 11.2 mm and a height of 4 mm. A tensile and compressive testing machine (HAIDA, China) was subsequently used for compression tests at a compression rate of 1 mm/min. The stress–strain curve of the hydrogel was calculated, and the compressive modulus was determined from the initial slope of the stress–strain curve within a strain range of 10% to 15%. Three replicate samples were tested for each condition.

The storage modulus (*G*′) and loss modulus (*G*″) were measured using a rheometer (Thermo Fisher Scientific, USA). The gel was positioned between 35-mm-diameter parallel plates with a 1-mm gap. Frequency scanning was performed at a constant strain of 1% and a frequency of 0.1 to 10 Hz. A viscoelastic analyzer (Rheolution, Canada) was used to analyze the viscoelasticity of the hydrogel samples at different time points. Two hundred microliters of hydrogel precursor solution was added to the matching culture mold and cured under blue light for 1 min to form a gel. At different time points, the matching culture mold containing the formed gel was placed on the machine, and the *G′* in the linear viscoelastic region was measured.

### Swelling and degradation behavior

The preweighed hydrogel (*W*_0_) was immersed in PBS (pH = 7.4) at 37 °C. The hydrogel sample was removed at predetermined time intervals, and the surface moisture was blotted with absorbent paper and weighed as *W*_t_. The swelling percentage of the hydrogel was calculated using the following formula:

$$\text{Swelling ratio}\;\left(\%\right)=\left(W_{t}-W_{0}\right)/W_{0}\times 100\%.$$ (1)

The preweighed hydrogel sample was immersed in 1 U/ml type I collagenase solution (Macklin, China) and incubated at 37 °C in a shaker at 20 rpm. The initial mass of the hydrogel was recorded as *W*_0_. At specified time intervals, the hydrogel sample was removed from the degradation solution, and the surface moisture was blotted with absorbent paper and weighed as *W*_t_. The residual mass percentage of the hydrogel was calculated using the following formula:

$$\text{Residual mass}\;\left(\%\right)=\left(W_{0}-W_{t}\right)/W_{0}\times 100\%.$$ (2)

### 5-Ethynyl-2′-deoxyuridine staining assay

Cell proliferation was detected using a 5-ethynyl-2′-deoxyuridine (EdU) detection kit (Beyotime, China). After counterstaining with Hoechst, the cells were observed and photographed using a fluorescence microscope. ImageJ software was used to quantify the number of EdU-positive cells.

### Live/dead cell staining assay

After 3 d of cell culture, the cells were washed twice with PBS and then stained with calcein-AM/propidium iodide (PI) solution (10 μM calcein-AM, 10 μM PI, and 10 ml of detection buffer) at 37 °C for 30 min. Fluorescence images were collected using a fluorescence microscope (Olympus, Japan), with excitation/emission filter pairs of 494/517 nm for calcein-AM (live cells) and 535/617 nm for PI (dead cells).

### Transwell cell migration assay

BMSCs and chondrocytes at a density of 3×10^4^ cells/ml were suspended in 200 μl of serum-free α-MEM and inoculated into the upper chamber of the Transwell insert. Two hundred microliters of 10% and 15% CMA hydrogel samples and 500 μl of α-MEM medium were added to the lower chamber. After being incubated for 48 h, the migrated cells were fixed with 4% paraformaldehyde (PFA; Beyotime, China) for 30 min, washed 3 times with PBS, and stained with 0.5% crystal violet solution (Beyotime, China) for 10 min. Images were taken using an inverted fluorescence microscope (Leica, Germany), and cell migration was quantitatively analyzed using ImageJ software.

### Cell Counting Kit-8 assay

On day 1 (D1), D3, and D7 of culture, Cell Counting Kit-8 (CCK-8) reagent (Beyotime, China) was added to each well at a ratio of 1:1 (medium to CCK-8) and incubated at 37 °C for 2 h. The absorbance of the supernatant was subsequently measured at 450-nm using a multifunctional microplate reader (Beyotime, China) to quantitatively evaluate the proliferation of BMSCs and chondrocytes.

### Reverse transcription–quantitative polymerase chain reaction

Total mRNA was extracted using the TransZol Up Plus RNA Kit (TransGen, China). cDNA was synthesized by reverse transcription using the PrimeScript RT Reverse Transcription Kit (TaKaRa, Japan). Reverse transcription–quantitative polymerase chain reaction (RT–qPCR) was performed using SYBR Green PCR Mix (Servicebio, China). Relative mRNA expression was calculated using the 2^(−ΔΔCt)^ method. All sequences of primers used in this study were purchased from Tsingke Biotech (China). The primer sequences are provided in the Supplementary Materials (Table S2).

### Animal experiment

Sixteen 8-week-old SD rats with an average body weight of 200 g were randomly divided into 4 groups (*n* = 4): the blank control group (Ctrl group), 15% CMA hydrogel group, BMSCs@CMA composite hydrogel group, and sham operation group (Sham group). Before the experiments, the BMSCs were cultured and adjusted to a concentration of 1 × 10^6^ cells/ml and uniformly mixed with 15% CMA hydrogel precursor solution to prepare the BMSCs@CMA composite hydrogel precursor solution for later use. All the rats were anesthetized by intraperitoneal injection, and the femoral trochlear surface of the knee joint was exposed under aseptic conditions. In the Sham group, only skin and joint capsule incisions were performed without cartilage defect creation, whereas full-thickness cylindrical cartilage defects (2.0 mm in diameter, 1.0 mm in depth) were created in the femoral trochlear groove using a sterile drill for the experimental groups. After modeling, no treatment was performed on the defect area in the Ctrl group; 15% CMA hydrogel precursor solution and preprepared BMSCs@CMA composite hydrogel precursor solution were accurately injected into the defect area in the 15% CMA group and BMSCs@CMA group, respectively, followed by 30-s of 405-nm ultraviolet irradiation to in situ photocrosslink and solidify the hydrogel. Routine feeding was performed after surgery, and the activity and incision condition were observed. The animals were sacrificed 12 weeks after surgery, and knee joint samples were collected for analysis.

### Gross observation

The International Cartilage Repair Society (ICRS) cartilage injury grading system was used to macroscopically evaluate cartilage repair. Specifically, the defect filling, tissue integration, and surface flatness of each sample knee joint were visually inspected.

### Micro-computed tomography

A SkyScan1275 micro-computed tomography (micro-CT) system (Bruker, Germany) was used to obtain detailed images of the rat knee joints, focusing on the knee joint cartilage, distal femur, and proximal tibia. The scanning parameters were as follows: a resolution of 18 μm, a rotation step of 0.20°, a scanning range of 180°, a voltage of 50 kV, and a current of 60 μA. Image reconstruction was completed using NRecon and Data Viewer software. Bone histomorphometric parameters were quantitatively analyzed using CT Analyzer software. Generation of 3D models was performed using CT vox software.

### Histological and immunohistochemical staining

The tissue samples were fixed with 4% PFA for 48 h and decalcified in EDTA decalcification solution for 6 weeks. All the samples were dehydrated with gradient ethanol and then embedded in paraffin for tissue sectioning (thickness of 10 μm). Hematoxylin–eosin (H&E) staining, safranin O/fast green (SO&FG) staining, and Masson staining were performed on the sections using the Servicebio (China) staining protocol. The sections were also incubated with primary and secondary antibodies for immunofluorescence (IF) and immunohistochemical (IHC) analysis. The full specific evaluation dimensions, itemized scoring standards, and score range of the modified O’Driscoll scoring system adopted in this study have been fully provided in the Supplementary Materials (Table S3).

### RNA sequencing and data processing

Total RNA was directly extracted from cell samples using the TransZol Up Plus RNA Kit (TransGen, China). The corresponding RNA library was constructed by APExBIO Technology (China), and the transcripts were analyzed by high-throughput sequencing. Sequencing data were processed using DESeq software to identify genes whose expression was significantly different (*P* < 0.05 and |log_2_ [fold change]| > 1). In addition, Kyoto Encyclopedia of Genes and Genomes (KEGG) pathway enrichment analysis, Gene Ontology (GO) analysis, and protein–protein interaction (PPI) network were performed using advanced algorithms in the R software package.

### Molecular docking and molecular dynamics simulation

The proteins used for molecular docking were rhCOL17 (Protein Data Bank [PDB]: 8k4x) and TGF-β1 (PDB: 4kv5). Protein–protein docking was performed via the HDOCK server, with docking score as the criterion to screen the optimal binding conformation. The selected complex model was subjected to 100-ns molecular dynamics (MD) simulations using GROMACS 2024. Briefly, topology files were generated with the AMBER14 force field and SPC216 water model; a TIP3P solvent box with a 1.0-nm boundary was built, and Na^+^/Cl^−^ ions were added for charge neutralization. The system underwent NVT (300 K, V-rescale thermostat) and NPT (1 bar, Berendsen barostat) equilibration, followed by production MD with a 2-fs leap-frog timestep, LINCS bond constraint, 1.0-nm interaction cutoff, and PME for long-range electrostatics; trajectories were recorded every 10 ps. Post-simulation, the Molecular Mechanics/Generalized Born Surface Area (MM/GBSA) and Molecular Mechanics/Poisson–Boltzmann Surface Area (MM/PBSA) approaches were applied to compute total binding free energy (BFE) and decompose residue-level energetic contributions, and conformations with favorable binding energies were visualized via PyMOL 2.4.

### Statistical analysis

All experimental data were obtained from at least 4 parallel samples in each detection group, and the results are expressed as the mean ± standard deviation (SD). One-way analysis of variance (ANOVA) was performed using GraphPad Prism 10.1.2 software to evaluate the statistical significance of differences between groups. The rules for indicating statistical significance are as follows: *P < 0.05, **P < 0.01, ***P < 0.001, and ****P < 0.0001.

## Results

### Preparation and characterization of the CMA hydrogel

**Fig. 2.**
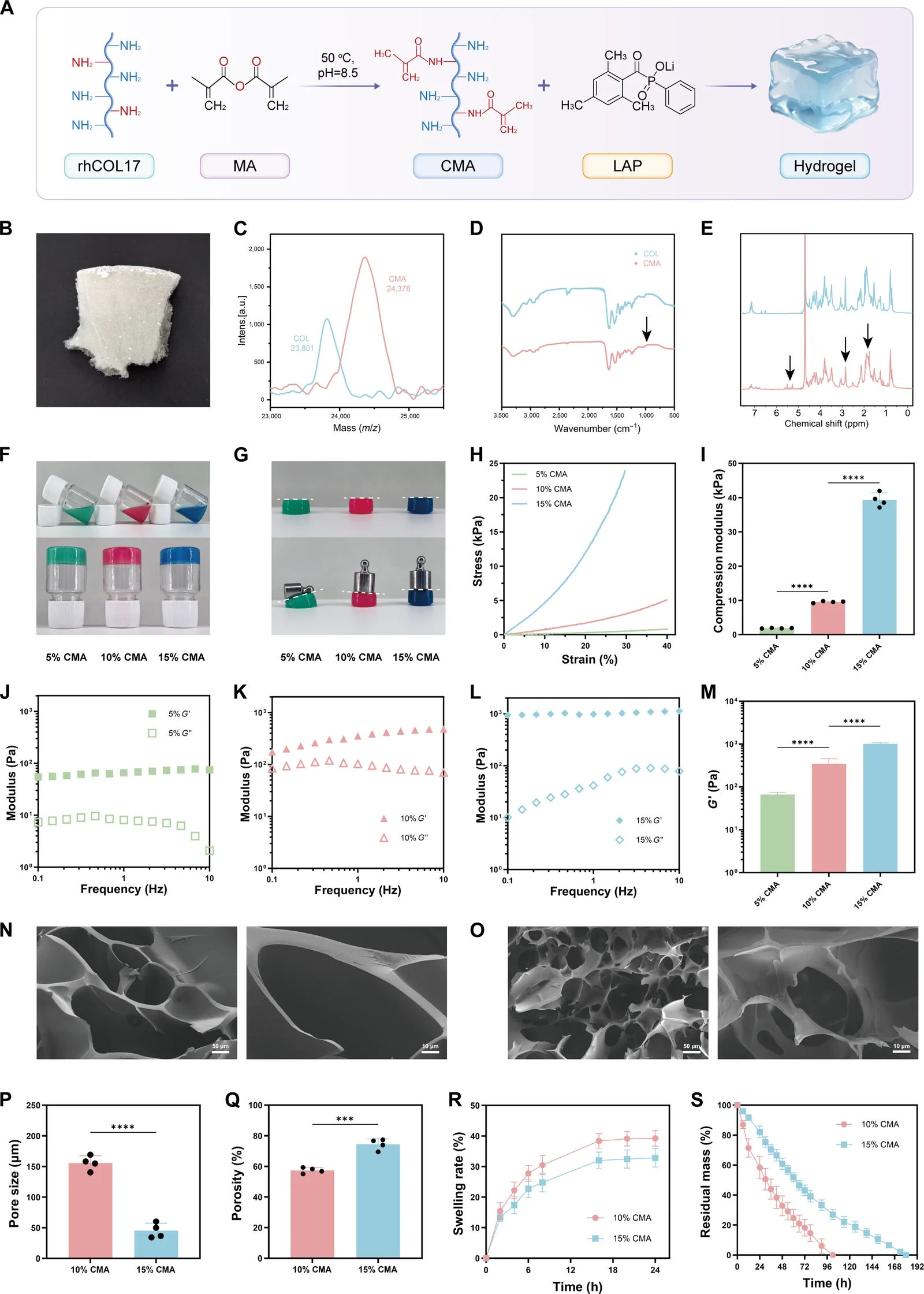
Synthesis and characterization of CMA. (A) Schematic of the synthesis of CMA by modifying rhCOL17 with MA and the preparation of the hydrogel with the photoinitiator LAP. (B) Photograph of lyophilized CMA. (C) Time-of-flight mass spectrometry (TOF-MS) analysis. (D) Fourier transform infrared (FTIR) spectrum. (E) ^1^H nuclear magnetic resonance (^1^H NMR) spectrum. (F) Photographs of the CMA precursor solutions and the corresponding photocrosslinked hydrogels. (G) Photographs of the macroscopic compression tests of the CMA hydrogels. (H) Stress–strain curves. (I) Statistical analysis of the compressive modulus. (J to L) Rheological frequency sweep curves for storage modulus (*G′*) and loss modulus (*G″*). (M) Statistical analysis of the *G*′. (N and O) Scanning electron microscopy (SEM) images. (P) Statistical analysis of pore size. (Q) Statistical analysis of porosity. (R and S) Swelling ratio and in vitro degradation rate curves over time. COL denotes rhCOL17, and CMA denotes methacrylated rhCOL17. Data are shown as the mean ± standard deviation (SD). Statistical significance: *P < 0.05, **P < 0.01, ***P < 0.001, ****P < 0.0001 for all pairwise group comparisons.

In this study, photosensitive methacryloyl groups were successfully grafted onto the collagen backbone through the reaction between MA and the amino groups of rhCOL17, resulting in the synthesis of a photocrosslinkable CMA material. Furthermore, the photoinitiator LAP (lithium phenyl [2,4,6-trimethylbenzoyl] phosphate) was introduced, and intermolecular crosslinking of CMA was induced under 405-nm ultraviolet irradiation, ultimately resulting in the formation of a collagen-based hydrogel system (Fig. 2A). After freeze-drying, the CMA produced a white, fluffy sponge-like porous solid with a loose texture, completely retaining the 3D network structure and providing a favorable structural basis for the subsequent construction of the hydrogels (Fig. 2B).

The TOF-MS analysis confirmed the chemical modification results (Fig. 2C). The molecular weight of CMA increased from 23,801 to 24,378 Da, indicating that methacryloyl groups were successfully introduced into the rhCOL17 molecules. FTIR analysis revealed (Fig. 2D) that while it retained all the characteristic absorption peaks of rhCOL17 (e.g., —C=O— stretching vibration at 1,616 cm^−1^) [27], CMA exhibited a unique new peak in the range of 950 to 1,000 cm^−1^, corresponding to the out-of-plane bending vibration of the propylene double bond (—C=C—) [28]. This strongly proves the successful modification of MA without destroying the original core secondary structure of collagen. ^1^H NMR analysis revealed (Fig. 2E) that new characteristic peaks appeared at 1.8, 2.8, and 5.5 ppm, attributed to the methyl group (—CH_3_), amino group (—NH_2_), and propylene double bond (—C=C—) in the methacryloyl group, respectively [26]. The graft efficiency of MA was calculated to be 33.64% by quantitatively analyzing the shift changes in peaks related to lysine residues [29]. This moderate functionalization level is crucial for ensuring the introduction of sufficient photocrosslinking sites while maximally retaining the natural conformation and biologically active sites of rhCOL17, laying a foundation for the construction of hydrogels with both mechanical stability and biological functionality.

After the CMA precursor solutions of different concentrations (5%, 10%, and 15%) were mixed with the photoinitiator LAP, the propylene double bonds underwent rapid photopolymerization under 405-nm ultraviolet, converting the liquid precursor into a solid hydrogel within seconds (Fig. 2F). This rapid in situ curing capability endows the hydrogel material with excellent application potential for repairing articular cartilage defects [30]. Macroscopic compression tests intuitively demonstrated the effect of the CMA concentration on mechanical stability (Fig. 2G). As indicated by the dashed line, the initial heights of the 3 hydrogels were consistent without weight loading. Under a 10-g load, the 5% CMA hydrogel collapsed markedly, and the 10% CMA showed slight deformation, whereas the 15% CMA remained almost intact, indicating that the 15% CMA hydrogel exhibited stronger structural stability and compressive bearing capacity under compressive loading. Stress–strain curves and statistical analysis revealed that the compressive modulus of the CMA hydrogel increased in a concentration-dependent manner (Fig. 2H and I). The compressive strength of the 15% CMA (39.32 kPa) was approximately 20 times greater than that of the 5% CMA (1.86 kPa). This mechanical enhancement stems from the increased crosslinking density caused by the increased polymer concentration, enabling the hydrogel network to disperse and bear loads more effectively, which is crucial for maintaining the structural integrity of the defect filler in a dynamically loaded joint environment [31]. Rheological tests further revealed the viscoelasticity of the CMA hydrogel (Fig. 2J to L). All concentrations of CMA exhibited typical gel behavior, with the *G*′ higher than the *G*″, and both increased slowly with increasing frequency, indicating a relatively stable structure of CMA. Compared with those of the 5% CMA, the elasticity and viscosity of the 10% and 15% CMA were considerably greater, and the 15% CMA had the highest *G*′ of 1,013.62 Pa (Fig. 2M).

Analysis of the above material characterization results revealed that the 5% CMA hydrogel has marked defects, such as extremely poor compressive performance and insufficient viscoelasticity, because the polymer concentration is too low and the crosslinked network structure is too loose. The 5% CMA hydrogel collapsed structurally under a slight load, could not provide stable mechanical support for cell growth, and failed to meet the basic structural requirements of scaffold materials for the dynamic mechanical microenvironment of joints; thus, this concentration of hydrogel was excluded from subsequent experiments. All further material characterization, cell- and animal-related experiments, were systematically conducted on 10% and 15% CMA hydrogels with superior mechanical properties and structural stability.

SEM images revealed that both the 10% (Fig. 2N) and 15% (Fig. 2O) CMA hydrogels exhibited highly interconnected and uniformly distributed porous 3D network structures. As the concentration increased from 10% to 15%, the average pore size of the CMA decreased from 155.70 ± 11.88 μm to 45.33 ± 12.06 μm (Fig. 2P), and the average porosity increased correspondingly from 57.36% ± 1.84% to 74.40% ± 3.57% (Fig. 2Q). The smaller pore size and higher porosity of 15% CMA provide an ideal spatial environment for cell migration, proliferation, and free diffusion of nutrients and metabolic waste [32]. Swelling experiments (Fig. 2R) revealed that the swelling equilibrium of the CMA hydrogel was reached within 16 h in PBS, and the equilibrium swelling ratio of 15% CMA (32.84% ± 3.00%) was lower than that of 10% CMA (39.21% ± 2.54%). This is attributed to the dense network formed by the high crosslinking density, which limits water penetration. Moderate swelling helps the hydrogel fit closely with surrounding tissues after implantation while reducing the interface gap between the material and the cartilage defect area, resulting in the formation of a stable local microenvironment for subsequent cartilage tissue regeneration [33]. In vitro enzymatic degradation experiments revealed (Fig. 2S) that the 15% CMA hydrogel took approximately 180 h to completely degrade in type I collagenase solution, which was longer than the 102 h required for 10% CMA. Degradation kinetics indicated that 15% CMA could provide a longer in vivo residence time, matching the long physiological cycle required for cartilage repair and facilitating continuous mechanical support and biological signals for tissue regeneration.

### The CMA hydrogel enhances BMSC proliferation, recruitment, and chondrogenic differentiation in vitro

**Fig. 3.**
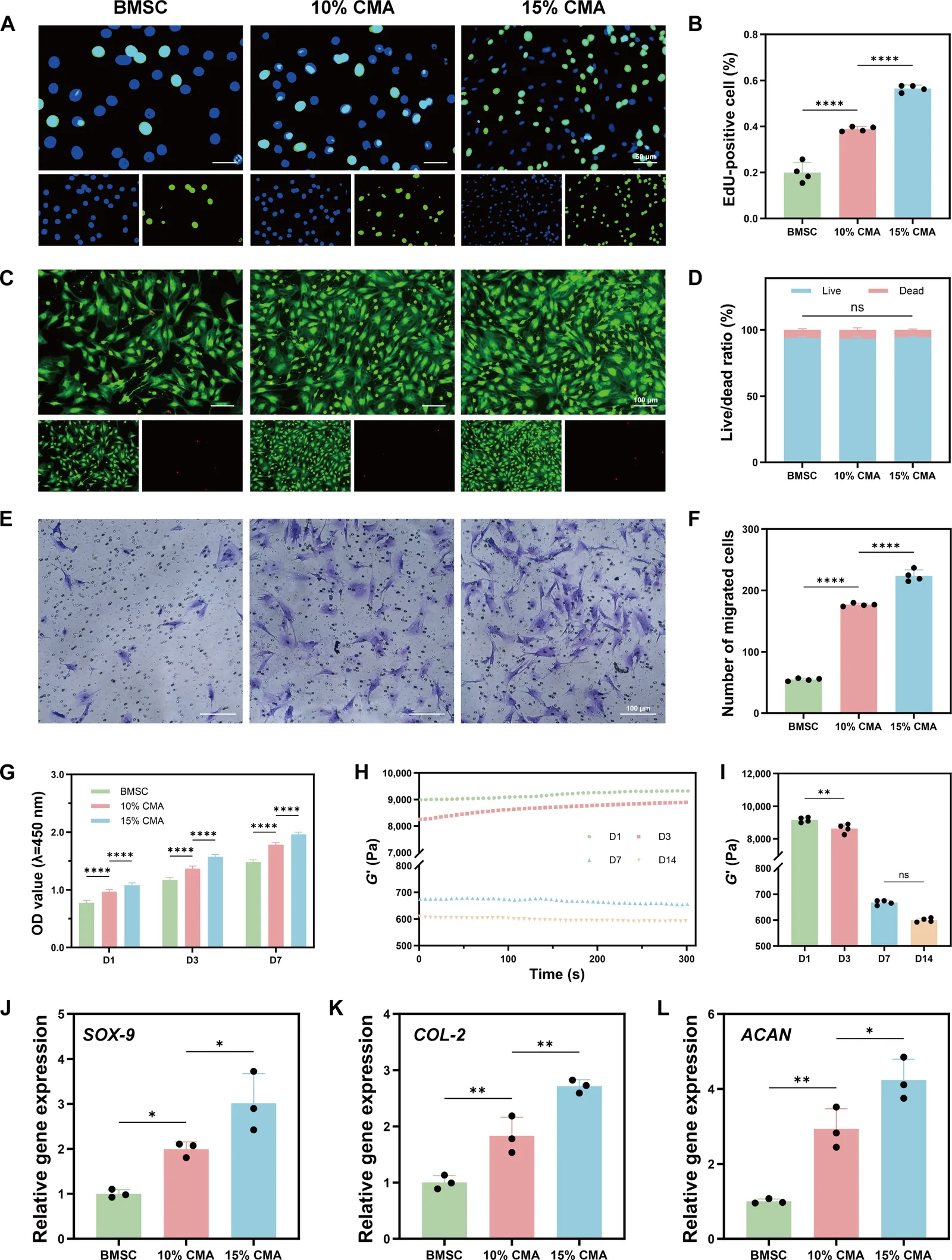
In vitro proliferation, viability, migration, and chondrogenic differentiation of BMSCs cultured with the CMA hydrogel. (A) EdU proliferation assay. (B) Statistical analysis of the percentage of EdU-positive cells. (C) Live/dead cell staining assay. (D) Statistical analysis of the live/dead cell ratio. (E) Transwell cell migration assay. (F) Statistical analysis of the number of migrated cells. (G) Statistical analysis of the results of the CCK-8 assay. (H) Time-dependent curve of the *G*′ of the 15% CMA hydrogel during culture. (I) Statistical analysis of the *G*′ of the 15% CMA hydrogel at different time points. (J to L) RT-qPCR verification of the relative expression levels of cartilage-specific genes (SOX9, COL2, and ACAN). Data are shown as the mean ± SD. Statistical significance: *P < 0.05, **P < 0.01, ***P < 0.001, ****P < 0.0001 for all pairwise group comparisons.

EdU proliferation assays (Fig. 3A and B) demonstrated that the proportion of EdU-positive BMSCs was significantly elevated in 10% and 15% CMA hydrogel groups relative to the Ctrl group cultured on conventional 2-dimensional (2D) culture plates (*P* < 0.05). These findings indicate that the 3D biomimetic microenvironment constructed by CMA is more conducive to the proliferation of BMSCs, ensuring the rapid recruitment and expansion of a sufficient number of functional cells inside the scaffold and in the defect area at the initial stage of cartilage defect repair, which is a necessary prerequisite for tissue regeneration [34]. Live/dead cell staining confirmed the excellent biocompatibility of CMA (Fig. 3C and D). After 3 d of culture, the proportion of live cells (green) in all 3 groups exceeded 95%, with sporadic dead cells (red). This finding demonstrated the non-cytotoxic nature of the CMA hydrogel, supporting its suitability as a cell carrier. Transwell migration assays revealed (Fig. 3E and F) that the CMA hydrogel had a chemotactic effect on BMSCs. Compared with cells cultured on standard 2D culture plates, the number of cells migrating to the lower chamber significantly increased in the 10% and 15% CMA groups (*P* < 0.05). These findings suggest that the CMA hydrogel can effectively recruit endogenous BMSCs to the cartilage defect site. CCK-8 assays further quantified the proproliferative effect of CMA on BMSCs (Fig. 3G). During the culture periods of 1, 3, and 7 d, the cell viability of the CMA groups was greater than the 2D Ctrl group, and the 15% CMA group showed the strongest proproliferative effect on D7, indicating that the 3D microenvironment provided by CMA can continuously support cell growth. Time rheological tests revealed the mechanical stability of the hydrogel in the culture environment (Fig. 3H and I). On D1 and D3, the *G*′ of the 15% CMA remained at a high level of 8,000 to 9,000 Pa; on D7 and D14, *G*′ decreased markedly to approximately 600 to 700 Pa. This trend indicates that the CMA hydrogel can provide a stable mechanical microenvironment for cells in the early stage and then undergo controllable degradation, resulting in sufficient space for newly generated tissue. RT–qPCR analysis revealed the ability of CMA to induce chondrogenic differentiation in BMSCs (Fig. 3J to L). After 7 d of culture in CMA hydrogel, the mRNA expression levels of cartilage-specific genes SOX9 (key transcription factor), COL2 (main matrix component), and ACAN (aggrecan) were significantly elevated in BMSCs of both the 10% and 15% CMA groups relative to the 2D Ctrl group, with the 15% CMA group showing higher gene expression than the 10% CMA group (*P* < 0.05). These findings prove that CMA not only is an inert scaffold but also can actively regulate stem cells fate and induce their differentiation into chondrocyte lineages.

### The CMA hydrogel supports chondrocyte phenotype and anabolic activity in vitro

**Fig. 4.**
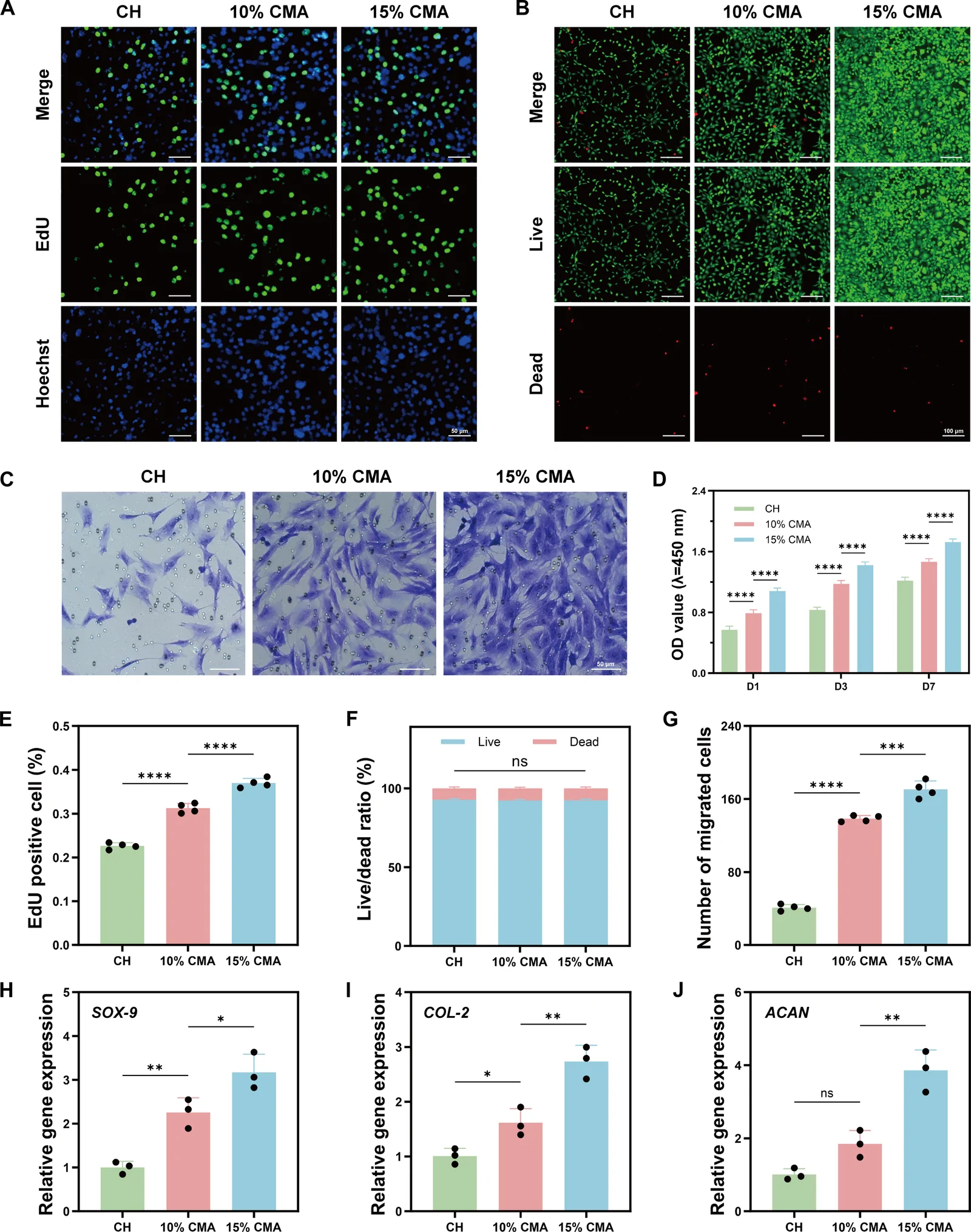
In vitro cytocompatibility, proliferation, migration, and phenotype maintenance of chondrocytes cultured with the CMA hydrogel. (A) EdU proliferation assay. (B) Live/dead cell staining assay. (C) Transwell cell migration assay. (D) Statistical analysis of the results of the CCK-8 assay. (E) Statistical analysis of the percentage of EdU-positive cells. (F) Statistical analysis of the live/dead cell ratio. (G) Statistical analysis of the number of migrated cells. (H to J) RT-qPCR verification of the relative expression levels of cartilage-specific genes (SOX9, COL2, and ACAN). CH denotes chondrocyte. Data are shown as the mean ± SD. Statistical significance: *P < 0.05, **P < 0.01, ***P < 0.001, ****P < 0.0001 for all pairwise group comparisons.

Similarly, compared with the 2D Ctrl group, the results of the EdU assays (Fig. 4A and E) confirmed that the CMA hydrogel substantially promoted the proliferation of primary chondrocytes, and the proportion of EdU-positive cells in the 15% CMA group was greater than that in the 10% CMA group (*P* < 0.05). Live/dead staining (Fig. 4B and F) assessed the cytocompatibility of CMA hydrogel. Live cells predominated and dead cells were sparse across all groups. Higher live-cell proportions in each group verified the favorable biological performance of CMA hydrogel. The results of the Transwell migration assays (Fig. 4C and G) revealed that compared with those in the 10% CMA group, the number of migrated cells in the 15% CMA group significantly increased (*P* < 0.05). Moreover, the results of the CCK-8 assays (Fig. 4D) illustrated that the OD value of the 15% CMA group at each time point was also significantly greater than that of the 10% CMA group (*P* < 0.05). Furthermore, the RT–qPCR results (Fig. 4H to J) demonstrated that the gene expression levels of SOX9, COL2, and ACAN in chondrocytes cultured in CMA were notably up-regulated, and the up-regulation amplitude of 15% CMA was significantly greater than that of 10% CMA (*P* < 0.05). These findings indicate that CMA can not only stably maintain the chondrocyte phenotype but also increase the activity of the synthesis of cartilage-specific matrix, which markedly affects chondrocytes, and that this biological regulatory effect is prominent at 15% CMA.

### The CMA hydrogel promotes cartilage defect repair in vivo

**Fig. 5.**
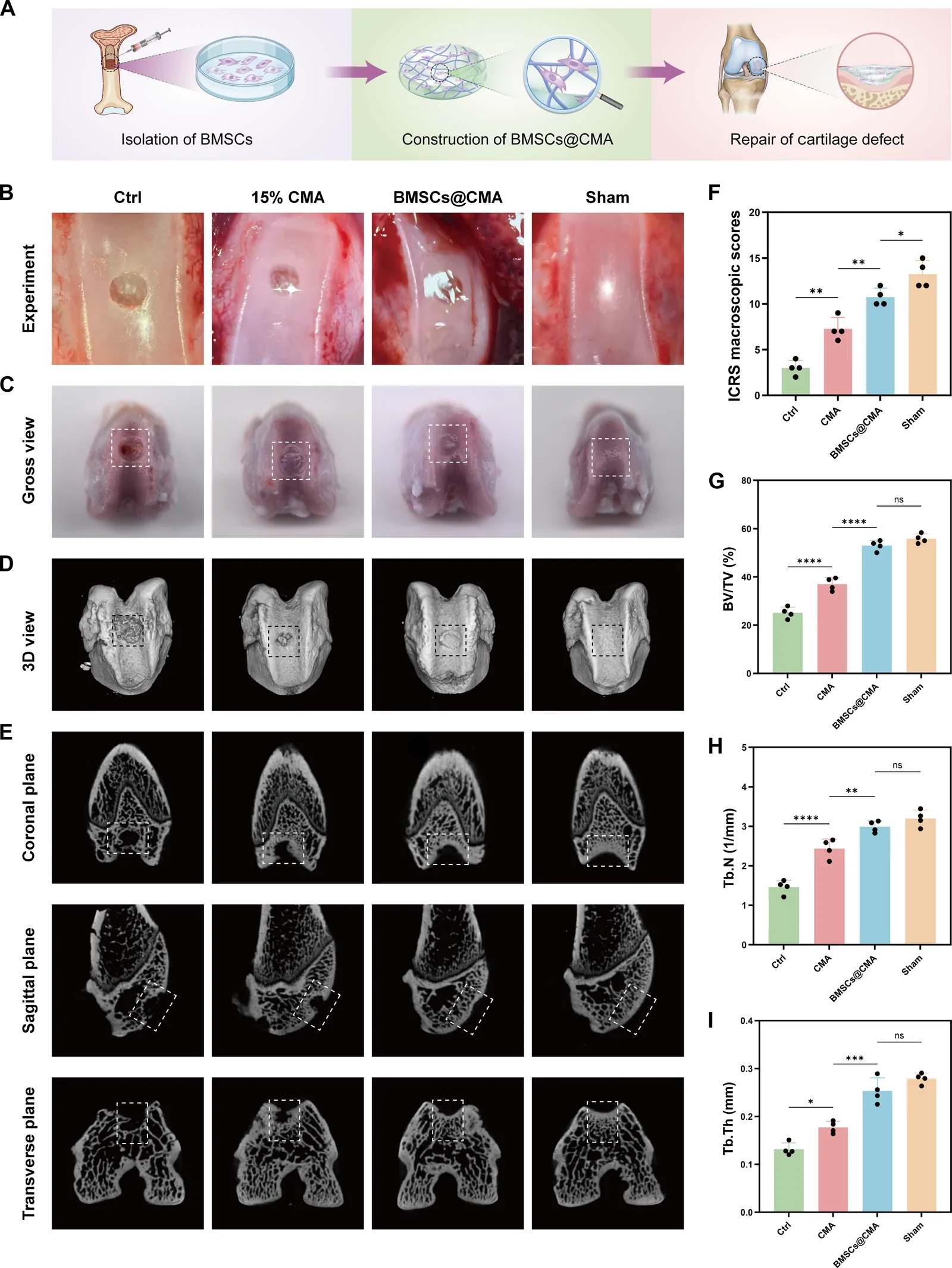
Evaluation of the in vivo repair effect of the BMSCs@CMA composite hydrogel on rat knee joint full-thickness cartilage defect. (A) Schematic of the in vivo experimental procedure. (B and C) Gross photographs of cartilage defect modeling and sample harvesting. (D and E) Micro-computed tomography (micro-CT) 3-dimensional reconstruction and 2-dimensional tomographic images of subchondral bone in the knee joint. (F) ICRS macroscopic scores of cartilage defect repair. (G to I) Quantitative analysis of subchondral bone morphometric parameters in the defect area (including bone volume fraction [BV/TV], trabecular number [Tb.N], and trabecular thickness [Tb.Th]). Data are shown as the mean ± SD. Statistical significance: *P < 0.05, **P < 0.01, ***P < 0.001, ****P < 0.0001 for all pairwise group comparisons.

To systematically evaluate the in vivo cartilage defect repair efficacy of the CMA hydrogel, a 15% CMA hydrogel with optimal mechanical and biological properties was selected as the carrier to encapsulate BMSCs, and the BMSCs@CMA composite hydrogel system was used to fill rat knee joint full-thickness cartilage defects (diameter 2.0 mm, depth 1.0 mm) created in the femoral trochlear groove (Fig. 5A and B). During the entire in vivo experiment, none of the animals developed surgical-site complications including infection, redness, swelling, or abnormal exudation; all exhibited normal joint activity. These observations indicated that the CMA hydrogel had favorable in vivo biocompatibility, that the implantation process was safe, and that no obvious local or systemic adverse reactions were induced (Figs. S1 and S2).

The samples were harvested 12 weeks after the cartilage defect model was established, and macroscopic observation revealed (Fig. 5C) that the defect in the Ctrl group was still clearly visible and only partially filled with fibrous tissue; the defect in the 15% CMA group was filled with repaired tissue, but the surface was uneven, whereas the defect area in the BMSCs@CMA group was almost completely filled, and the surface of the repaired tissue was smooth and integrated well with the surrounding native cartilage. Macroscopic scoring by the ICRS quantitatively confirmed that the synergistic effect of BMSCs and CMA could significantly improve the macroscopic repair effect of cartilage defects (*P* < 0.05; Fig. 5F).

To further evaluate the repair quality of the subchondral bone, micro-CT technology was used to analyze bone mass in the rat knee joints 12 weeks after surgery. 3D reconstruction and 2D tomographic images intuitively revealed the regeneration of subchondral bone (Fig. 5D and E), and the regeneration quality of the subchondral bone in each group markedly differed. The defect area in the Ctrl group was almost not filled with new bone tissue, and the gray value was low; partial filling was observed in the 15% CMA group, and the gray value recovered slightly, whereas the defect area in the BMSCs@CMA group formed continuous and complete bone filling, and the gray value was close to that of the surrounding normal bone tissue, indicating that the degree of mineralization of the new bone was close to the physiological level. Quantitative analysis of the region of interest further confirmed (Fig. 5G to I) that the bone volume fraction (BV/TV), trabecular number (Tb.N), and trabecular thickness (Tb.Th) in the BMSCs@CMA group were significantly greater than those in the Ctrl group and CMA group (*P* < 0.05), and there was no significant difference in any bone histomorphometric parameters between the BMSCs@CMA group and the Sham group. These findings indicate that the BMSCs@CMA composite hydrogel can not only promote cartilage layer regeneration but also effectively guide the reconstruction of subchondral bone, achieving integrated osteochondral repair with superior biomechanical function.

In summary, the results of in vivo experiments revealed that the 15% CMA hydrogel loaded with BMSCs resulted in optimal cartilage regeneration and subchondral bone reconstruction for the repair of cartilage defects through cooperative biological effects, providing a novel therapeutic strategy and an experimental basis with great potential for the regenerative repair of cartilage defects.

### Histological assessment of the CMA hydrogel in cartilage regeneration

**Fig. 6.**
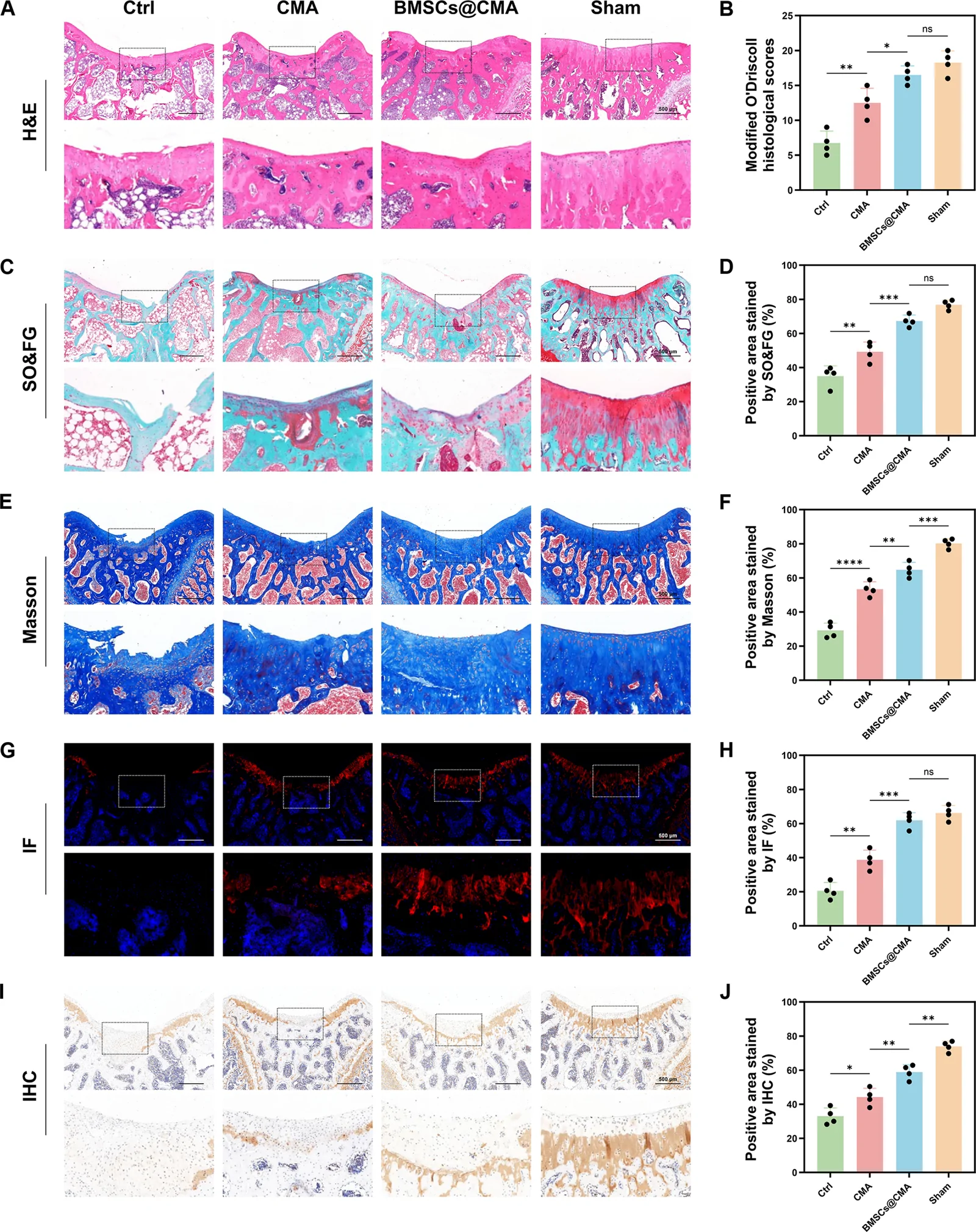
Histological and immunological analysis of the effects of the BMSCs@CMA composite hydrogel on repairing rat knee joint full-thickness cartilage defect. (A) Hematoxylin and eosin (H&E) staining. (B) Modified O'Driscoll histological scores. (C and D) Safranin O/Fast Green (SO&FG) staining and semiquantitative analysis of the positive area. (E and F) Masson staining and semiquantitative analysis of the positive area. (G and H) Immunofluorescence (IF) staining for COL2 and semiquantitative analysis of the positive area. (I and J) Immunohistochemistry (IHC) staining for COL2 and semiquantitative analysis of the positive area. Data are shown as the mean ± SD. Statistical significance: *P < 0.05, **P < 0.01, ***P < 0.001, ****P < 0.0001 for all pairwise group comparisons.

The histological staining results were used to systematically evaluate the structure and composition of the repaired tissue, and the effects of the repair were further quantified by statistical analysis. H&E staining revealed (Fig. 6A) that the defect area in the Ctrl group was filled only with disorderly fibrous tissue; although the 15% CMA group formed new tissue, the number of chondrocytes was small, and the morphology was atypical and lacked clear stratification, while the BMSCs@CMA group formed a stratified structure similar to that of the Sham group, with typical chondrocyte clusters. The modified O’Driscoll histological score [35] (Fig. 6B) further confirmed that the BMSCs@CMA group achieved significantly higher values than the Ctrl group and CMA group (*P* < 0.05), with no significant difference from that of the Sham group, indicating that the structural integrity of the repaired tissue was highly consistent with normal cartilage tissue. SO&FG staining (Fig. 6C) revealed that the BMSCs@CMA group presented strong safranin O positivity with massive deposition of glycosaminoglycans (GAGs), and the percentage of positively stained area (Fig. 6D) was comparable to that of the Sham group, which was significantly greater than that of the 15% CMA group (only weak positive staining) and the Ctrl group (no obvious positive staining) (*P* < 0.05). Masson staining (Fig. 6E) revealed that the collagen matrix did not form continuously in the Ctrl group; the 15% CMA group exhibited only a small amount of dispersed cartilage matrix, whereas the BMSCs@CMA group formed a continuous and orderly blue-stained collagen matrix layer with structural integrity close to that of the Sham group. Positive staining area statistics (Fig. 6F) revealed that the collagen matrix-positive ratio in the BMSCs@CMA group was significantly higher than that in the Ctrl group and CMA group (*P* < 0.05). Immunostaining analysis of COL2 further verified the synthesis of the cartilage-specific matrix. IF (Fig. 6G) and IHC (Fig. 6I) results consistently revealed that the expression of COL2 presented a marked intergroup gradient: almost no COL2 expression in the defect area of the Ctrl group (only nuclear fluorescence in IF, no dark brown staining in IHC); weak and scattered COL2 expression in the 15% CMA group (red fluorescence in IF, dark brown staining in IHC); the BMSCs@CMA group displayed robust COL2-positive signals. Quantitative analysis (Fig. 6H and J) further demonstrated that the COL2 positive-staining area ratio of the BMSCs@CMA group was comparable to that of the Sham group, indicating that the repaired tissue in this group had synthesized and deposited a large amount of COL2, possessing the key matrix characteristics of hyaline cartilage.

In summary, the results of multidimensional staining indicate that compared with the single 15% CMA group and the Ctrl group, the BMSCs@CMA composite hydrogel can effectively induce chondrocyte maturation, promote cartilage-specific matrix synthesis and tissue structure reconstruction, and achieve functional repair of cartilage defects.

### Mechanisms of cartilage regeneration by the CMA hydrogel

To explore the molecular mechanism by which the 15% CMA hydrogel modulates BMSCs to enhance their capacity for cartilage repair, a blank control group (the Ctrl group, in which BMSCs were routinely cultured under 2D conditions) and a 15% CMA hydrogel group (the CMA group, in which BMSCs were encapsulated in 15% CMA hydrogel for 3D culture) were established. Cells were collected after 7 d of culture for comparative RNA extraction and transcriptome sequencing analysis.

**Fig. 7.**
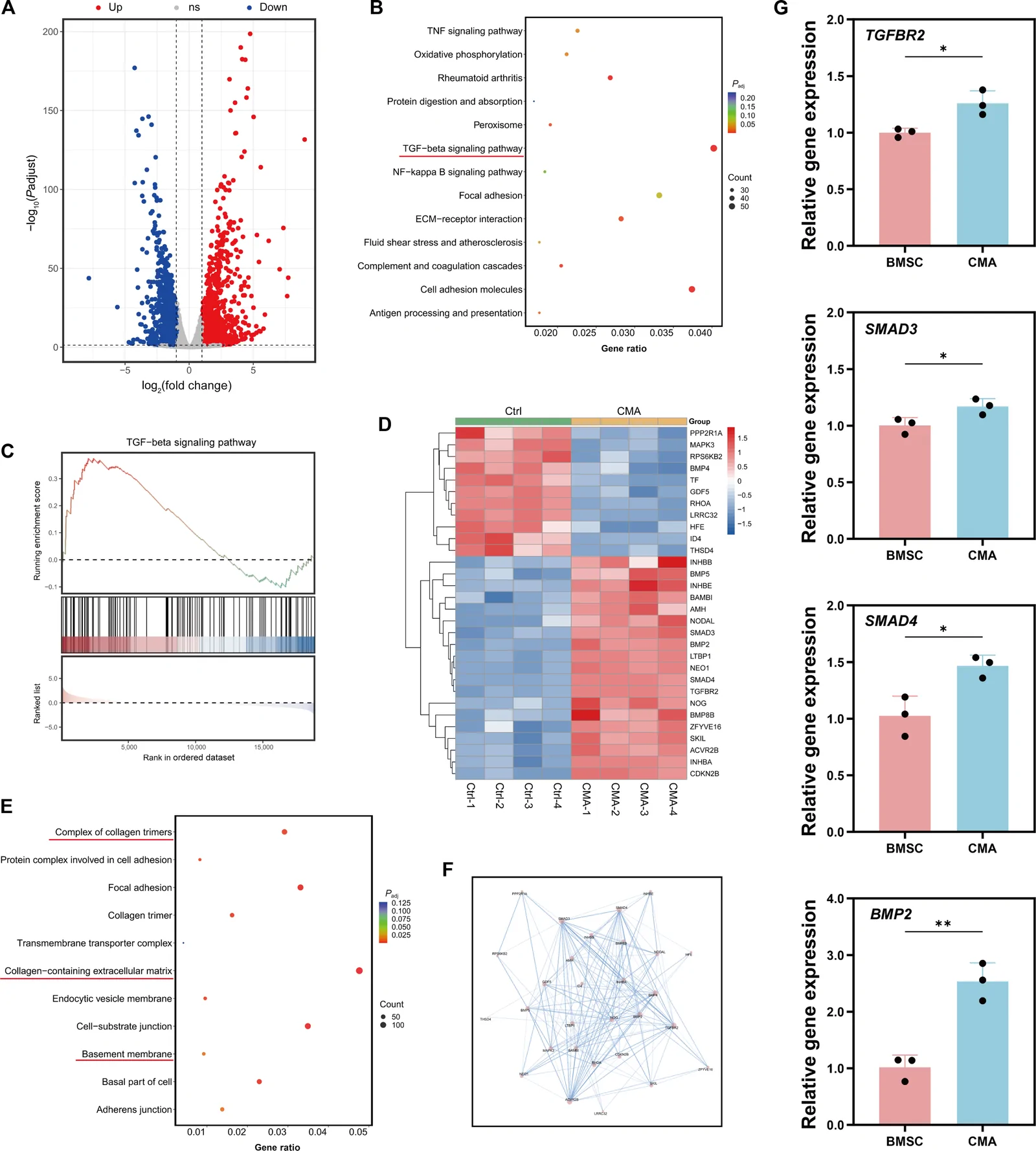
Transcriptome sequencing analysis of the molecular mechanism by which the CMA hydrogel promotes cartilage defect repair. (A) Volcano plot of differentially expressed genes (DEGs). (B) KEGG functional enrichment bubble plot. (C) Gene Set Enrichment Analysis (GSEA) based on the KEGG database for the TGF-β signaling pathway. (D) Heatmap analysis of genes related to the TGF-β signaling pathway. (E) GO cellular component (GO-CC) enrichment bubble plot. (F) Protein–protein interaction (PPI) network of the TGF-β signaling pathway. (G) RT-qPCR verification of the relative expression levels of core genes in the TGF-β signaling pathway (TGFBR2, SMAD3, SMAD4, and BMP2). Data are shown as the mean ± SD. Statistical significance: *P < 0.05, **P < 0.01, ***P < 0.001, ****P < 0.0001 for all pairwise group comparisons.

Volcano plot (Fig. 7A) analysis revealed 3,124 differentially expressed genes (DEGs) between the CMA group and the Ctrl group, including 1,349 up-regulated genes and 1,775 down-regulated genes, suggesting that the 3D culture microenvironment of the CMA hydrogel can reshape the gene expression profile of BMSCs. KEGG functional enrichment analysis of the DEGs (Fig. 7B) revealed that pathways such as chondrogenic differentiation, ECM interaction, and cell adhesion were markedly enriched, among which the TGF-β signaling pathway became the core regulatory pathway. As this classic TGF-β/SMAD signaling axis dominates BMSCs lineage specification, it facilitates chondrogenic differentiation and boosts the synthesis of cartilage-specific matrix; meanwhile, mechanical microenvironmental cues dynamically tune its activity to drive BMSCs into distinct chondrocyte subtypes [36–38]. Gene Set Enrichment Analysis (GSEA) based on the KEGG database (Fig. 7C) further confirmed that the TGF-β signaling pathway was markedly activated in the CMA group, and the synergistic differential expression of core genes in the pathway was the key driver of cartilage repair. The heatmap of DEGs in the TGF-β signaling pathway (Fig. 7D) revealed an expression pattern highly consistent with that of the cartilage repair phenotype. Among the DEGs, the key receptor TGFBR2, the core transcription factor SMAD3/4, and the superfamily member BMP2 of the pathway were up-regulated in the CMA group. GO cellular component (GO-CC) enrichment (Fig. 7E) analysis revealed that the enriched cellular components were involved mainly in collagen synthesis, cell adhesion, and basement membrane organization, which confirmed the cartilage specificity and structural integrity of the repaired tissue at the cellular structure level. The PPI network of the TGF-β signaling pathway (Fig. 7F) illustrates the interactions among core molecules, with TGFBR2, SMAD3, SMAD4, BMP2, BMP4, and BMP5 identified as major hubs. Through direct contacts, these hubs associate with collagen-regulatory factors and cell adhesion molecules, thereby coupling TGF-β signal transduction to both cartilage matrix synthesis and cell–matrix adhesion. RT–qPCR was used to verify the key genes screened by the transcriptome (Fig. 7G), and the results revealed that compared with those in the Ctrl group, the expression of core genes related to the TGF-β pathway (TGFBR2, SMAD3, SMAD4, and BMP2) in the CMA group was up-regulated, which was consistent with the transcriptome sequencing results.

In summary, the 15% CMA hydrogel facilitates activation of the TGF-β signaling pathway and modulates expression of core genes in the pathway, thereby promoting the differentiation of BMSCs into chondrocytes and the synthesis of cartilage-specific matrix, ultimately leading to functional repair of cartilage defects.

### Molecular interactions between rhCOL17 and TGF-β

**Fig. 8.**
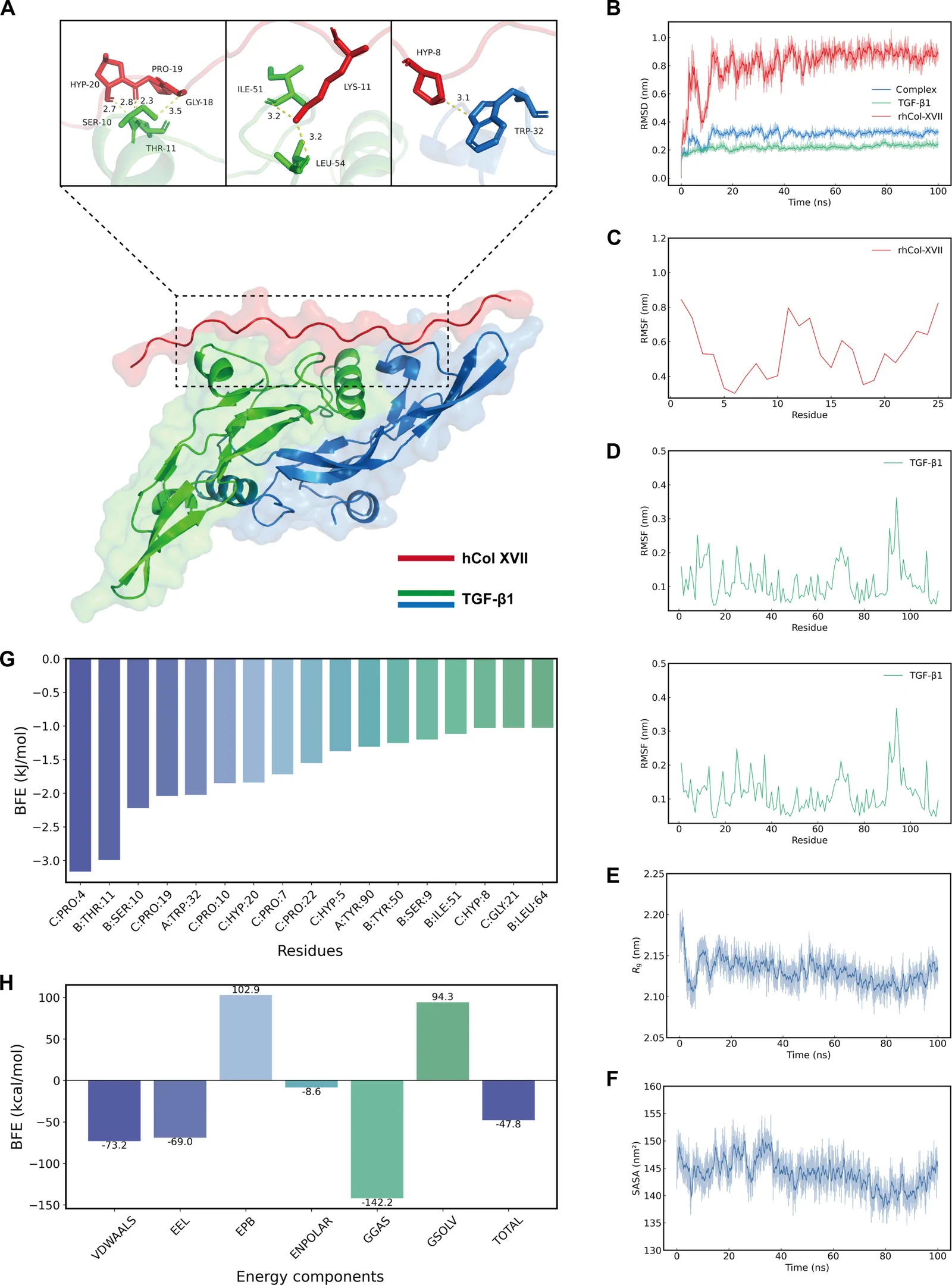
Molecular docking and molecular dynamics simulations of the interaction between rhCOL17 and TGF-β1. (A) Schematic of the molecular docking structure and key binding residue interactions. (B) Root-mean-square deviation (RMSD) curve during simulation. (C) Root-mean-square fluctuation (RMSF) curve of rhCOL17. (D) RMSF curves of 2 single chains of TGF-β1. (E) Radius of gyration (*R*_g_) curve of the rhCOL17/TGF-β1 complex. (F) Solvent-accessible surface area (SASA) curve of the rhCOL17/TGF-β1 complex. (G) Per-residue contribution analysis of the binding free energy (BFE). (H) Energy component analysis of the BFE.

To further elucidate the molecular basis for the activation of the TGF-β signaling pathway by the CMA hydrogel, we systematically verified the key molecular interactions between rhCOL17 and TGF-β1 through molecular docking and 100-ns all-atom MD simulation. The molecular docking results (Fig. 8A) revealed the specific binding interface between these 2 molecules. The core residues of rhCOL17 and TGF-β1 were anchored through hydrogen bonds and hydrophobic interactions, suggesting that rhCOL17 can intervene in the active conformation or intracellular localization of TGF-β1 through direct physical binding, providing initial molecular structural evidence for the activation of the TGF-β signaling pathway. MD simulations (Fig. 8B to F) revealed that the root mean square deviation of the rhCOL17/TGF-β1 complex remained at 0.2 to 0.4-nm throughout the 100-ns simulation, the root mean square fluctuation of residues in the core binding region was markedly reduced, and the radius of gyration and solvent accessible surface area fluctuated stably, collectively confirming that the complex had a convergent conformation, compact structure, and no dissociation under a physiological environment. Energetic analysis (Fig. 8G and H) further verified the specificity and stability of the complex structure from a thermodynamic perspective. The residue-level BFE decomposition results were highly consistent with the key interaction sites identified by molecular docking. The global total BFE was −47.8 kcal/mol, among which van der Waals and electrostatic interactions were the main energy sources driving binding. In summary, this study verified the high specificity and strong stability of the binding between rhCOL17 and TGF-β1 from 3 dimensions, namely, structural recognition, dynamic conformation, and thermodynamic energy, providing evidence of microscopic structural molecular interactions for CMA hydrogel-mediated cartilage repair via activation of the TGF-β signaling pathway.

## Conclusion

Taken together, our in vitro experiments and the rat osteochondral defect repair model demonstrated that CMA hydrogel possessed potent chondrogenic induction capacity and promoted effective cartilage regeneration. Nevertheless, several inherent limitations of the present research should be acknowledged before these findings can be translated into preclinical large-animal studies and clinical practice [39]. As typical small rodents, rats possess thinner articular cartilage tissue, stronger spontaneous self-repair ability of cartilage defects, faster ECM metabolism rate, and lower joint mechanical load compared with large mammals such as rabbits, sheep, and pigs as well as human beings [40]. The satisfactory repair effect observed in rat models cannot be completely duplicated under the physiological and mechanical conditions of large-animal knee joints and human weight-bearing joints [41]. Besides, the physicochemical properties including mechanical modulus and degradation rate of CMA hydrogel are only adapted to the microenvironment of rat articular cavity; targeted parameter optimization is indispensable to match the mechanical demand of human normal cartilage and the degradation cycle of human cartilage repair. Therefore, the research conclusions obtained from rodent models need to be treated cautiously during subsequent large-animal trials and clinical translational research [42].

In addition to the interspecies differences of animal models mentioned above, another obvious deficiency of this study lies in the relatively short in vivo observation period [43]. We only evaluated the short-term histological repair status of defective cartilage within several weeks after implantation, lacking long-term tracking detection of hydrogel in vivo degradation dynamics, neocartilage matrix long-term stability, and joint functional recovery [44]. Combined with the limitations and potential clinical transformation demand summarized above, our subsequent research plan mainly includes 3 aspects [45]: conducting large-animal osteochondral defect repair experiments to verify the in vivo safety and long-term repair effect of CMA hydrogel; prolonging the in vivo observation cycle to evaluate the long-term durability of regenerated cartilage tissue; and optimizing the formulation and mechanical properties to narrow the gap between bench research and clinical application, so as to steadily promote the translational progress of the CMA hydrogel [46].

Furthermore, clinical traumatic and degenerative osteochondral defects form a persistent inflammatory microenvironment [47], wherein accumulated TNF-α, IL-1β, and other pro-inflammatory factors induce chondrocyte injury, restrain endogenous chondrogenesis, and aggravate matrix degradation to hinder cartilage repair [48]. The interconnected porous structure of the CMA hydrogel enables physical adsorption of excess inflammatory cytokines in lesion sites, ameliorating local inflammatory stress and forming a favorable microenvironment for cell survival and chondrogenic differentiation. Combined with the verified cartilage repair performance in this study, the CMA hydrogel possesses promising prospects for the treatment of inflamed osteochondral defects [49]. For subsequent research, we will quantitatively evaluate the cytokine adsorption capacity of the CMA hydrogel under varying inflammatory conditions. Moreover, its porous skeleton can be used as a delivery platform for sustained release of anti-inflammatory agents, thereby optimizing pathological joint microenvironment and further boosting the clinical translational value in osteochondral regeneration [50].

In summary, this study pioneers the application of rhCOL17 for articular cartilage defects, addressing the inherent limitations of conventional collagen-based hydrogels. We successfully engineered an injectable and photocrosslinkable hydrogel based on rhCOL17 and, after systematic screening, identified the 15% CMA formulation as the optimal candidate. This formulation retains the native core structure and bioactivity of rhCOL17, while exhibiting mechanical properties well-suited to the joint microenvironment, including a 3D porous network, moderate swelling, controlled degradation kinetics, and rapid in situ photocrosslinking capability that fulfills clinical requirements for minimally invasive delivery. In vitro, the CMA hydrogel demonstrated excellent biocompatibility and markedly enhanced the proliferation, migration, and chondrogenic differentiation of both BMSCs and chondrocytes. In a rat knee joint full-thickness cartilage defect model, the BMSCs-laden 15% CMA composite hydrogel achieved robust osteochondral integrated repair. The regenerated tissue closely resembled native hyaline cartilage in macroscopic morphology, subchondral bone mineralization, histological architecture, and deposition of GAGs and COL2. Mechanistically, we demonstrate that rhCOL17 directly binds to TGF-β1 and activates the canonical TGF-β/SMAD signaling pathway, thereby orchestrating the chondrogenic differentiation of BMSCs and enabling an integrated “material scaffold-seed cell-biological signal” repair paradigm. Collectively, this work not only establishes a novel, minimally invasive, and mechanically adaptable strategy for functional cartilage defect repair but also lays a critical foundation for expanding the cross-tissue applications of rhCOL17 in regenerative tissue engineering.

## Data Availability

The data that support the findings of this study are available from the corresponding authors (J.G., Z.L., Y.S., and X.H.) upon reasonable request.
