# Supplementary material for: An Injectable and Photocrosslinkable Hydrogel Based on Recombinant Human Collagen Type XVII Promotes Osteochondral Regeneration by Activating the TGF β/SMAD Signaling Pathway
Source: Biomater Res. 2026 Sep 14;30:0412. doi: 10.34133/bmr.0412 (PMC13572800; doi:10.34133/bmr.0412)
Supplement: Supplementary file 1 [file bmr.0412.f1.docx]

**Supplementary Materials**

**Figure S1** After 12 weeks of implantation, hematoxylin-eosin (HE) staining demonstrated intact tissue structure and normal cellular morphology in the heart, liver, spleen, lung and kidney of rats from all groups. No pathological injuries such as tissue necrosis, extensive inflammatory cell infiltration, tissue congestion or pathological degeneration were detected in visceral organs. These results confirm that both CMA hydrogel and BMSCs@CMA display satisfactory long-term in vivo biosafety with no evident systemic organ toxicity after implantation.

**Figure S2** At 12 weeks of implantation, hematological analysis indicated that all tested biomarkers, including routine blood parameters (RBC, HGB, WBC, PLT), liver function indices (ALT, AST, TBIL, ALB), serum lipid profiles (TC, HDL, LDL, TG) and renal function markers (BUN, CREA, CYS C), stayed within normal physiological ranges among all experimental groups. This suggests that long-term implantation of CMA hydrogel and BMSCs@CMA exerts no functional impairment to the hematopoietic system, liver, kidney or lipid metabolism in rats.

**Table S1** Amino acid sequence of the human type XVII collagen.

| **10** | **20** | **30** | **40** | **50** |
| --- | --- | --- | --- | --- |
| **YVWSHPQFEK** | **GSPGPKGDMG** | **SPGPKGDRGF** | **PGTPGIPGPL** | **GHPGPQGPKG** |
| **60** | **70** | **80** | **90** | **100** |
| **QKGSVGDPGM** | **EGPGEKGERG** | **PGVPGSVGPK** | **AAGEPGPHGP** | **GSSGSPGPQG** |
| **110** | **120** | **130** | **140** | **150** |
| **PPGPVGLQGL** | **RGEVGLPGVK** | **GDKGPMGPPG** | **PKGDQGEKGP** | **PGPPGPPGPK** |
| **160** | **170** | **180** | **190** | **200** |
| **GDQGPPGPRG** | **HQGEQGLPGF** | **SGPPGPPGPQ** | **GPKGDKGDPG** | **VPGALGIPGP** |
| **210** | **220** | **230** | **240** |  |
| **PGQKGEMGTP** | **GPKGDRGPAG** | **PPGHPGPPGP** | **RGHKGEKGDK** | **GDQHHHHHH** |

**Table S2** Primer sequences used for RT-qPCR.

| **Gene (rat)** | **Primer sequences (5’-3’)** | |
| --- | --- | --- |
| SOX9 | F: AAGTCGGTGAAGAATGGGCA | R: GTCGGTGGACCCTGAGATTG |
| COL2 | F: GGCCAGGATGCCCGAAAATTA | R: CCCTCTCTCCCTTGTCACCAC |
| ACAN | F: CTGGGTGGATGCAGAGAGAC | R: TTGGTTTGGACGCCACTTCT |
| TGFBR2 | F: GAGAAGCCGCAGGAAGT | R: AGTGAAGCCGTGGTAGGT |
| SMAD3 | F: AGGGCTTTGAGGCTGTCTACC | R: TGCTGGTCACTGTCTGTCTCC |
| SMAD4 | F: GGTCCGTAGGTGGAATAGCC | R: AATCCAGCACGGGGTTTCTT |
| BMP2 | F: GAAGCCAGGTGTCTCCAAGAG | R: GTGGATGTCCTTTACCGTCGT |
| GAPDH | F: GAAGGTCGGTGTGAACGGAT | R: CCCATTTGATGTTAGCGGGAT |

**Table S3** The Modified O’Driscoll Score for Histological Assessment of Cartilage Repair.

| **Item** | **Score (Points)** |
| --- | --- |
| Hyaline cartilage | ≤20% (0), 21%–40% (2), 41%–60% (4), 61%–80% (6), 81%–100% (8) |
| **Structural characteristics** |  |
| Surface irregularity | Severe disruption and fibrillation (0), fissures (1), smooth and intact (2) |
| Structural integrity | Severe lack of integration (0), slight disruption including cysts (1), normal (2) |
| Thickness | ≤50% (0), 51%–100% (1), 100% of normal adjacent cartilage (2) |
| Bonding to adjacent cartilage | Not bonded (0), bonded at 1 end/partially at both ends (1), bonded at both ends of graft (2) |
| Free from degenerative cellular changes | Moderate hypocellularity, >25% clusters (0); slight hypocellularity, ≤25% chondrocyte clusters (1); normal cellularity, no clusters (2) |
| Free from degenerative changes in adjacent cartilage | Severe hypocellularity, slight staining (0); mild or moderate hypocellularity, slight staining (1); normal cellularity, mild clusters, moderate staining (2); normal cellularity, no clusters, normal staining (3) |
| Reconstitution of subchondral bone | ≤50% (0), 51%–100% (1), complete reconstitution (2) |
| Bonding of repair cartilage to de novo subchondral bone | ≤50% (0), 51%–100% (1), complete and uninterrupted (2) |
| Safranin O staining | ≤0% (0), 41%–80% (1), >80% (2) |
